# Autonomic dysfunction and treatment strategies in intracerebral hemorrhage

**DOI:** 10.1111/cns.14544

**Published:** 2024-02-19

**Authors:** Kaijiang Kang, Kaibin Shi, Jiexin Liu, Na Li, Jianwei Wu, Xingquan Zhao

**Affiliations:** ^1^ Department of Neurology Beijing Tiantan Hospital Capital Medical University Beijing China; ^2^ China National Clinical Research Center for Neurological Diseases Beijing China; ^3^ Center of Stroke Beijing Institute for Brain Disorders Beijing China; ^4^ Research Unit of Artificial Intelligence in Cerebrovascular Disease Chinese Academy of Medical Sciences Beijing China

**Keywords:** autonomic dysfunction, autonomic nervous system, intracerebral hemorrhage, sympathetic hyperactivity, vagus nerve stimulation

## Abstract

**Aims:**

Autonomic dysfunction with central autonomic network (CAN) damage occurs frequently after intracerebral hemorrhage (ICH) and contributes to a series of adverse outcomes. This review aims to provide insight and convenience for future clinical practice and research on autonomic dysfunction in ICH patients.

**Discussion:**

We summarize the autonomic dysfunction in ICH from the aspects of potential mechanisms, clinical significance, assessment, and treatment strategies. The CAN structures mainly include insular cortex, anterior cingulate cortex, amygdala, hypothalamus, nucleus of the solitary tract, ventrolateral medulla, dorsal motor nucleus of the vagus, nucleus ambiguus, parabrachial nucleus, and periaqueductal gray. Autonomic dysfunction after ICH is closely associated with neurological functional outcomes, cardiac complications, blood pressure fluctuation, immunosuppression and infection, thermoregulatory dysfunction, hyperglycemia, digestive dysfunction, and urogenital disturbances. Heart rate variability, baroreflex sensitivity, skin sympathetic nerve activity, sympathetic skin response, and plasma catecholamine concentration can be used to assess the autonomic functional activities after ICH. Risk stratification of patients according to autonomic functional activities, and development of intervention approaches based on the restoration of sympathetic‐parasympathetic balance, would potentially improve clinical outcomes in ICH patients.

**Conclusion:**

The review systematically summarizes the evidence of autonomic dysfunction and its association with clinical outcomes in ICH patients, proposing that targeting autonomic dysfunction could be potentially investigated to improve the clinical outcomes.

## Graphical abstract

The review summarizes the autonomic dysfunction in intracerebral hemorrhage from the aspects of potential mechanisms, clinical significance, assessment, and treatment strategies, providing convenience for future clinical practice and research.

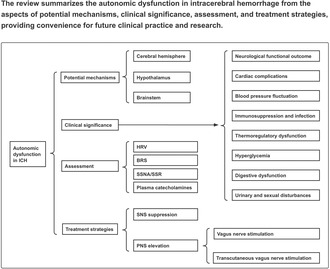

## 1 INTRODUCTION

Stroke is a leading cause of death and disability worldwide. Intracerebral hemorrhage (ICH) accounts for approximately 10%–30% of all strokes with higher rates in Asian and African countries.^1^, ^2^, ^3^ Compared with ischemic stroke, ICH has a higher mortality rate and worse clinical outcome but a lack of effective treatment available at present.^1^, ^4^

As a dreadful injury, ICH is often complicated with dysfunction of autonomic nervous system (ANS) and sympathetic‐parasympathetic unbalance, mainly manifested as elevated activity of sympathetic nervous system (sympathetic hyperactivity).^5^, ^6^, ^7^, ^8^ Autonomic dysfunction after stroke had been indicated to be associated with poor outcome and various complications, including cerebrocardiac syndrome, blood pressure elevation and variability, immunosuppression and infection, thermoregulatory dysfunction, and hyperglycemia.^5^, ^6^, ^7^, ^9^, ^10^ Actually, post‐stroke autonomic dysfunction had been supposed to be an independent predictor of poor neurological functional outcome, cardiac complications, blood pressure fluctuation, infection, and stroke recurrence.^5^, ^6^, ^11^, ^12^, ^13^ Also, studies suggested that modulation of ANS function, such as blocking sympathetic activity and stimulating parasympathetic activity can promote the recovery of neurological function of stroke patients.^14^, ^15^, ^16^, ^17^ Therefore, targeting the ANS for risk stratification of patients according to autonomic functional activities, development of intervention approaches based on the restoration of sympathetic‐parasympathetic balance, and more precise management on these basis, would potentially improve clinical outcomes of ICH patients.

With primary and series of secondary injuries, ICH is more likely to increase the activation level of sympathetic nervous system leading to autonomic dysfunction compared with other types of cerebrovascular disease. However, previous studies on autonomic dysfunction in stroke mainly focused on ischemic stroke and subarachnoid hemorrhage, while the investigations on ICH were insufficient. This review article summarizes the autonomic dysfunction in ICH from the aspects of potential mechanisms, clinical significance, assessment, and treatment strategies, so as to provide convenience for future clinical practice and research.

## 2 POTENTIAL MECHANISMS

**FIGURE 1:**
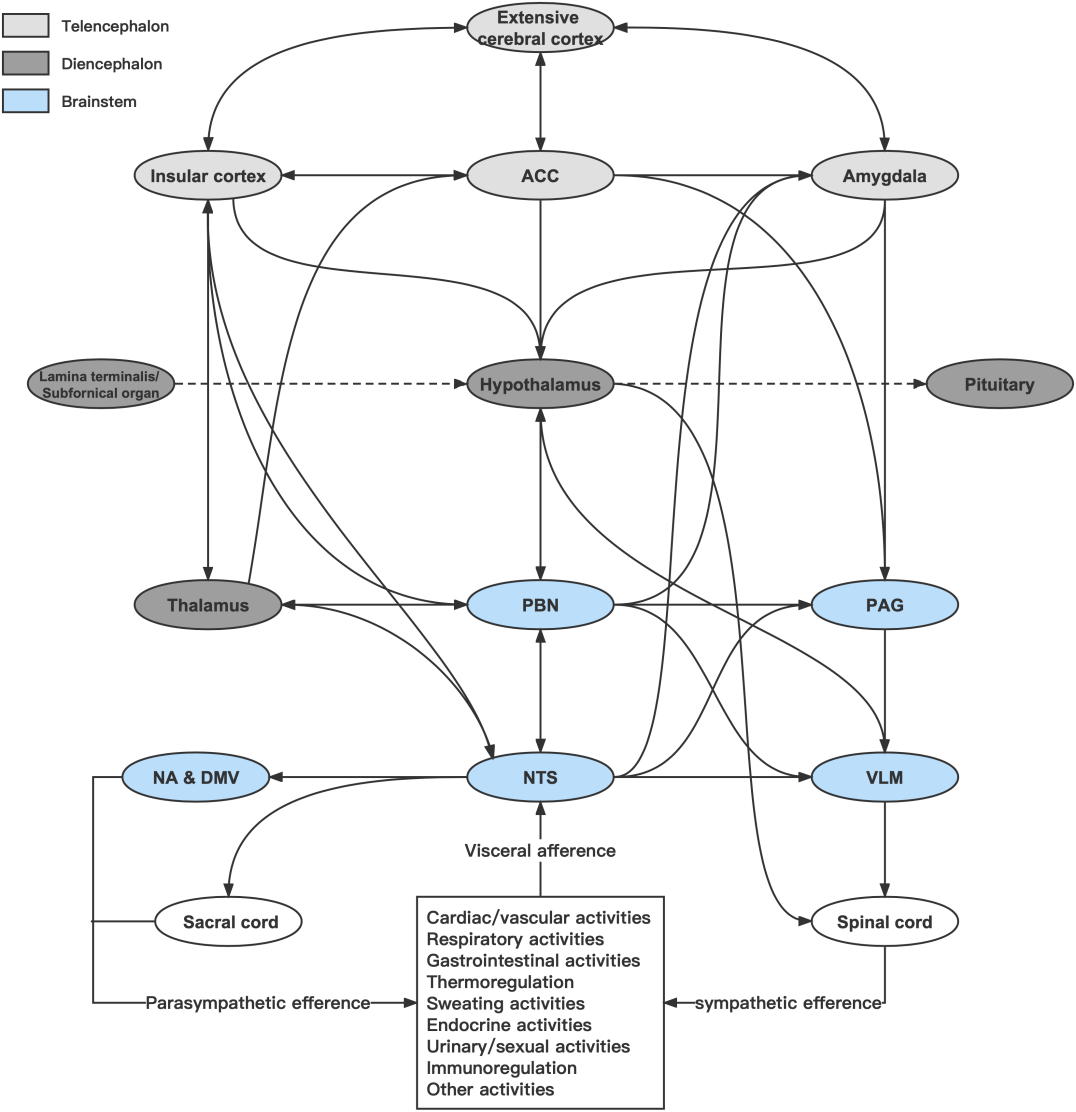
Diagram of central autonomic network. The diagram was drawn mainly based on the current researches to our best knowledge. ACC, anterior cingulate cortex; DMV, dorsal motor nucleus of the vagus; NA, nucleus ambiguus; NTS, nucleus of the solitary tract; PAG, periaqueductal gray; PBN, parabrachial nucleus; VLM, ventrolateral medulla.

The central autonomic network (CAN) is an integral component of an internal regulation system through which the brain receives viscerosensory inputs (relayed on the nucleus of the tractus solitarius) and humoral inputs (relayed through the circumventricular organs), and controls sympathetic and parasympathetic activities, neuroendocrine and other essential activities for survival.^18^, ^19^ It includes the cerebral cortex (especially insular cortex), amygdala, hypothalamus (especially paraventricular nucleus), autonomic nuclei in the brain stem (especially rostral ventrolateral medulla, nucleus of the tractus solitarius, periaqueductal gray matter and parabrachial complex), and the lateral horn of the spinal cord.^18^, ^19^, ^20^, ^21^ The CAN has the characteristics of mutual interconnection, condition‐dependent activity, and neurochemical complexity. Under normal conditions, the sympathetic nervous system (SNS) and parasympathetic nervous system (PNS) coordinate to regulate various physiological and metabolic activities of the body.^18^ The diagram of CAN mainly based on the current research to our best knowledge is shown in Figure 1.

When the primary and secondary injuries of ICH affect the above anatomical structures, it may cause autonomic dysfunction or sympathetic‐parasympathetic unbalance. Additionally, studies regarding the hemorrhage positions in human patients have suggested that hemorrhage in certain locations of the brain is more prevalent to associate autonomic dysfunction.

### 2.1 Cerebral hemisphere

The insular cortex, anterior cingulate cortex, and amygdala regulate high‐level autonomic control, and their injury can lead to autonomic disorder symptoms of varying degrees and systems.^9^, ^18^, ^22^ The insular cortex is the primary interoceptive cortex, which receives and integrates visceral and somatic sensory information from the thalamus, and controls both sympathetic and parasympathetic outputs with projection mainly to the lateral hypothalamic area.^9^, ^18^, ^20^ The anterior cingulate cortex receives afferent inputs mainly from the medial thalamic nuclei, interconnects with the insular cortex, and initiates autonomic responses associated with pain, motivation and goal‐directed behavior.^21^, ^23^ The amygdala has widespread connections with the hypothalamus and brainstem (particularly the periaqueductal gray and the medullary reticular formation), and initiates autonomic and neuroendocrine responses, which are essential for the expression of emotional responses.^18^, ^19^, ^22^, ^24^

It is indicated that ICH involving the insular cortex tends to be complicated with autonomic dysfunction and associated with worse short‐term outcomes independent from involved laterality or cardiac dysfunction.^6^, ^25^ The bilateral insular cortexes have been indicated to regulate sympathetic activity in humans and animal models, which may be the source of upstream tonic sympathetic inhibition.^26^, ^27^, ^28^ Hence, the hemorrhagic injury of the pathway from the insular cortex to downstream sympathetic centers may attenuate or eliminate the tonic inhibition from the insular cortex, resulting in excessive sympathetic output and activity.^26^, ^27^, ^28^

Extensive hemispheric hemorrhage that involves unilateral or bilateral hemispheres of the brain is often complicated with autonomic dysfunction. The underlying mechanism may be that diffuse injury results in the separation of one or more brain regions from each other, especially the disconnection of cortical autonomic inhibitory centers (such as the insular cortex and anterior cingulate cortex) with the diencephalon and brainstem centers.^26^, ^29^, ^30^

### 2.2 Hypothalamus

Hypothalamus is the subcortical center of ANS, of which the anterior area is the representative area of parasympathetic nerves, while the posterior area is the representative area of sympathetic nerves. The paraventricular nucleus (PVN) of the hypothalamus has been supposed as one of the most important autonomic control centers playing essential roles in autonomic and neuroendocrine regulation.^31^ The autonomic nuclei of the hypothalamus (including PVN), contain mixed populations of neurons that control specific subsets of preganglionic sympathetic and parasympathetic neurons. The PVN has been found to receive signals from the subset neurons of lamina terminalis and subfornical organ outside the blood–brain barrier and responsive to circulating angiotensin II, and other neurohormones, and send descending projections directly to sympathetic preganglionic nuclei in the spinal cord, or through an indirect pathway to other key autonomic nuclei, including the nucleus of the solitary tract (NTS) and rostral ventrolateral medulla (RVLM).^32^ The subset neuronal populations in PVN also express receptors for a variety of neurohormones and neurotransmitters, mainly including angiotensin II, neuropeptide Y, and vasopressin, all of which can regulate sympathetic activity.^33^ Therefore, the CAN works as an immediate brain warning system that responds to various emergency circumstances and is involved in the pathophysiological changes associated with various brain injuries.^34^

Studies had demonstrated hypothalamic injury on diffusion tensor imaging (DTI) in the case series of ICH concurrent with paroxysmal sympathetic hyperactivity.^35^, ^36^ Also, hematoma extension into the third or fourth ventricles had been indicated to cause autonomic dysregulation and associated adverse outcomes, which was presumably caused by the damage of ANS structures existing in the hypothalamus or brainstem surrounding the third or fourth ventricles, such as paraventricular nucleus, periaqueductal gray (PAG), and other circumventricular autonomic nucleus and fibers.^19^, ^37^ Intracerebral hematoma breaking into the ventricles usually causes the disturbance of cerebrospinal fluid (CSF) circulation and thus produces a local pressure effect contributing to the reduction of local cerebral blood flow in surrounding tissues.^38^ In addition, degraded blood cells may induce a series of immune and inflammatory reactions, which can result in secondary injury and edema of periventricular tissue.^39^

### 2.3 Brainstem

The autonomic structures in the brainstem mainly include NTS, ventrolateral medulla (VLM), dorsal motor nucleus of the vagus (DMV), nucleus ambiguus (NA), parabrachial nucleus (PBN), and PAG.^18^, ^40^ The NTS is the first relay station for visceral afferent information, transmitting information from baroreceptors, chemoreceptors, and cardiac, pulmonary, and gastrointestinal afferents, to the PBN, PAG, thalamus, hypothalamus, and amygdala.^18^, ^40^, ^41^, ^42^ The VLM is essential for the regulation of heart rhythm and blood pressure. The adrenergic and glutaminergic neurons in rostral VLM provide the tonic excitatory output for sympathetic preganglionic neurons that innervate resistance vessels in peripheral organs.^40^, ^43^ The GABAergic and noradrenergic neurons in the caudal VLM neurons mediate a variety of cardiovascular reflexes, which are involved in normal cardiac activity and various heart diseases.^40^, ^43^ The NTS and RVLM contain a network of respiratory and circulatory neurons, which play a critical role in the control of respiratory and circulatory activities under various circumstances.^18^, ^19^, ^40^ The DMV receives information from the NTS, and the projecting fibers from the preganglionic parasympathetic neurons forming the main component of the vagus nerve, regulating the vagovagal reflexes, and controlling gastrointestinal motility and secretion.^41^, ^42^ The NA receives exciting information from the baroreceptor‐sensitive neurons in NTS, and the efferent fibers project to the cardiac ganglia, conducting the major inhibitory control of the heart.^18^, ^43^ As an interface between the diencephalon and brain stem, the PAG has extensive connections with the spinal cord, brain stem, diencephalon, and cortex, playing critical roles in autonomic function (including the regulation of the circulatory, respiratory, urinary, reproductive, and thermoregulatory systems), pain control, and behavioral responses to threatening stimuli.^44^, ^45^ The PBN mainly receives visceral information from the NTS, nociceptive and thermoreceptive information from the spinal cord, and transmits them to the thalamus, hypothalamus, and amygdala. The PBN is also involved in regulating gustatory, salivary, gastrointestinal, cardiovascular, respiratory, osmotic, and thermoregulatory activities.^18^, ^40^, ^46^ Harboring a variety of autonomic nuclei and connective fibers, the brainstem is the primary center of autonomic control and regulation.^47^ Brainstem stroke has been demonstrated to be complicated with autonomic dysfunction and excessive sympathetic activities.^48^, ^49^, ^50^

## 3 CLINICAL SIGNIFICANCE

### 3.1 Neurological functional outcome

Autonomic dysfunction had been indicated to be associated with poor outcome after acute ischemic and hemorrhagic stroke.^5^, ^6^, ^51^, ^52^, ^53^, ^54^ Qu et al. compared the heart rate variability (HRV) of 122 ICH patients within 7 days from symptom onset and 122 matched controls and demonstrated that HRV was impaired significantly within 14 days after ICH, which was independently associated with 3‐month poor neurological outcomes.^7^ A post hoc analysis of ATACH‐2 (Antihypertensive Treatment in Intracerebral Hemorrhage 2) trial demonstrated that average heart rate real variability within the initial 24 h were independently associated with hematoma expansion at 24 h and unfavorable functional outcome at 90 days in acute ICH.^55^ A systematic review with retrieval of 19 significant studies had also demonstrated the association of HRV with poor functional outcome and mortality in ICH and subarachnoid hemorrhage.^56^ Sykora et al. compared the Baroreflex sensitivity (BRS) of 45 ICH patients within 72 h from symptom onset with 38 control subjects, and found that BRS decreased significantly in patients with acute ICH, and the decreased BRS was demonstrated to be an independent predictor of short‐term (10 days) poor neurological outcome.^6^ They also found a significant correlation between BRS and relative brain edema and a nonsignificant trend between hemorrhage enlargement and BRS.^11^ The proposed mechanism may be that autonomic dysfunction and shift to sympathetic hyperactivity with subsequent blood pressure fluctuation have worsened the course of the acute phase due to the formation of brain edema, hematoma enlargement, and possibly also to cerebral hypoperfusion.^11^ The central autonomic network is imperative for maintaining appropriate cerebral blood flow and cerebral function under normal conditions.^57^, ^58^ Sympathetic hyperactivity was suggested to be associated with leukocytosis, various proinflammatory cytotoxic changes, disturbance of cerebral circulation, and damage of the blood–brain barrier, which may promote secondary injury, such as cerebral ischemia and brain edema.^59^, ^60^ Therefore, targeting the autonomic nervous system for risk stratification and outcome prediction may help identify high‐risk ICH patients who may benefit from neuromodulation and additional management.^6^, ^51^

### 3.2 Cardiac complications

There exist complex connections between the brain and heart, and abnormal impulses from the brain may derange the structural or rhythmic abnormalities of the heart through the mediation of ANS.^12^ Reduced HRV and impaired BRS after stroke had been linked to higher risk of coronary ischemia, myocardial infarction, myocardial injury, arrhythmias, and sudden cardiac death, which is also called cerebrocardiac syndrome.^10^, ^12^, ^61^, ^62^ The main pathophysiological mechanism is assumed to be post‐stroke functional and structural alterations in the central autonomic network, with subsequent dysregulation of normal neural cardiac control.^12^

Increasing stroke severity was closely related to progressive global autonomic dysfunction, decrease in parasympathetic tone, and progressive shift toward sympathetic dominance, which put patients at increased risk of cardiac complications and poor outcome. Also, it is indicated that there may be lateralization of sympathetic and parasympathetic cardiac function.^12^, ^22^, ^62^, ^63^, ^64^ Sympathetic hyperactivity is likely to be associated with the prefrontal cortex, anterior cingulate cortex, left amygdala, right anterior insular cortex, and left posterior insular cortex.^22^, ^63^, ^64^, ^65^, ^66^ The high National Institutes of Health Stroke Scale (NIHSS), insular cortex involvement, presence of intraventricular hemorrhage, and hydrocephalus had been suggested to be associated with cardiac autonomic dysregulation after stroke.^67^, ^68^

### 3.3 Blood pressure fluctuation

Blood pressure dysregulation, manifested as blood pressure elevation and fluctuation, has been frequently reported in acute stroke, especially hemorrhagic stroke (up to 80% of cases), which was also indicated to be relevant to adverse outcomes.^6^, ^61^ Dysfunction of blood pressure regulation post‐stroke is time‐dependent, which can occur immediately after acute stroke and is gradually relieved over time.^69^ Sykora et al. analyzed the relationship between blood pressure and BRS in 83 subjects (45 patients with acute ICH and 38 control subjects) and demonstrated that ICH patients had significantly decreased BRS and increased blood pressure variability, which was proposed as an independent predictor of short‐term outcome after ICH.^6^

Autonomic disturbance (particularly baroreflex dysfunction) has been supposed to be the main cause of blood pressure variability after stroke, which may be associated with various pathophysiological mechanisms, including preexisting hypertension, activation of the renin‐angiotensin‐aldosterone axis, and stress response.^6^, ^61^, ^70^ It is generally manifested as sympathetic hyperactivity and parasympathetic underactivity causing transient blood pressure elevation and instability.^6^, ^9^, ^61^, ^69^, ^70^ However, orthostatic hypotensive response with sympathetic underactivity has also been reported in acute stroke.^71^

Increased blood pressure after acute ICH increases the risk of hematoma enlargement and poor outcome. However, there was no consistent result on whether early intensive blood pressure lowering can reduce the risk of hematoma enlargement and poor outcome in two randomized controlled trials (INTERACT2 and ATACH‐II).^72^, ^73^ Recently published INTERACT3 (the third Intensive Care Bundle with Blood Pressure Reduction in Acute Cerebral Hemorrhage Trial) indicated that the implementation of a care bundle protocol for intensive blood pressure lowering and management of hyperglycemia, pyrexia (likely signs of autonomic dysfunction), and abnormal anticoagulation within several hours of symptom onset resulted in improved functional outcome for patients with acute ICH.^74^ These trials (including the ongoing INTERACT4) mainly explore the target systolic blood pressure level, magnitude, and timing of blood pressure lowering, but lack of investigation on the specific types of antihypertensive drugs due to the regional differences in the availability of blood pressure lowering agents.^72^, ^73^ Considering that the main pathophysiological mechanism of elevated blood pressure in the acute phase of ICH is the sympathetic hyperactivity, inhibiting sympathetic activity may be more appropriate in lowering blood pressure in acute ICH.^6^, ^61^ Actually, the heterogeneity has been indicated in the efficacy and safety of various blood pressure lowering agents. Patients who received α‐ and β‐ blockers tend to have better outcomes from intensive blood pressure lowering compared with other agents, which suggests that α‐ and β‐blockers may benefit from inhibiting the sympathetic hyperactivity that initiates and aggravates the hypertensive response but remains to be further investigated.^75^

### 3.4 Immunosuppression and infection

Patients with ICH are often at high risk of developing stroke‐associated pneumonia and other infectious complications during acute hospitalization, which are common causes of adverse outcomes, and can increase the length of hospital stay and economic burden.^76^, ^77^ Immunosuppression frequently occurs after a stroke, which is supposed to be associated with the increased risk of infectious comorbidities in stroke patients.^78^, ^79^, ^80^, ^81^ Under normal conditions, the immune system and nervous system interact to maintain physiological stability and balance. Severe neurological disorders can disrupt this balance and cause various changes in both systems contributing to immune suppression and increased risk of infections.^79^, ^82^

Although the underlying mechanisms remain elusive, sympathetic hyperactivity associated with central nervous system injury and activation of the hypothalamus–pituitary–adrenal gland axis have been generally believed as the causes of post‐stroke immunosuppression.^79^, ^81^, ^83^, ^84^, ^85^ Sykora et al had demonstrated that autonomic shift with decreased BRS was independently associated with infections during the first 5 days of hospital stay after ICH, and during the first 7 days of hospital stay after ischemic stroke.^13^, ^86^ A recently published research established a connection between the sympathetic stress response, tissue innervation, and T‐cell exhaustion and indicated that the sympathetic hyperactivity can accelerate the depletion of T cells in chronic infections and cancers by secreting norepinephrine acting on the adrenergic receptor β1 on the surface of CD8 + T cells.^87^

### 3.5 Thermoregulatory dysfunction

Fever is one of the common complications after stroke (especially hemorrhagic stroke) associated with poor outcome.^88^, ^89^, ^90^ The incidence of fever is higher in patients with severe stroke and those complicated with intraventricular hemorrhage.^88^, ^89^, ^91^ Post‐stroke fever includes both infectious and non‐infectious fevers, and the main cause of non‐infectious fever is supposed to be thermoregulatory dysregulation (also called central hyperthermia), which is characterized by rapid onset and termination of high fever, remarkable temperature variation, and high mortality.^92^

The main mechanism of post‐stroke thermoregulatory dysregulation has been supposed to be autonomic dysfunction with sympathetic hyperactivity. The sympathetic skin response amplitude had been indicated to be significantly suppressed in hemispheric infarction compared with controls.^93^, ^94^ Swor et al. demonstrated that HRV was a potential marker of autonomic dysfunction and is associated with subsequent fever development in patients with ICH.^95^

### 3.6 Hyperglycemia

Hyperglycemia occurs frequently in patients with acute stroke, which has been indicated to be associated with poor functional outcome and mortality in both diabetic and non‐diabetic patients.^96^, ^97^, ^98^ Stress hyperglycemia secondary to acute stroke, usually assessed by stress hyperglycemia ratio, is characterized by transient hyperglycemia, and has been indicated to better predict mortality, poor neurological outcome, and infectious complications.^99^, ^100^, ^101^ Stress hyperglycemia has also been reported to be associated with early hematoma enlargement and perihematomal edema aggravation in patients with ICH.^98^, ^101^, ^102^, ^103^, ^104^

Post‐stroke stress hyperglycemia has been supposed to be associated with increased sympathetic activity and secretion of cortisol and norepinephrine, contributing to increased hepatic glucose output and insulin resistance.^105^, ^106^ Sykora et al. demonstrated an association between stress hyperglycemia and decreased BRS in non‐diabetic patients, which indicated that post‐stroke hyperglycemia may be the result of autonomic shift toward sympathetic hyperactivity.^106^

### 3.7 Digestive dysfunction

Digestive dysfunction (including dysphagia, stress ulcer, gastrointestinal bleeding, or fecal incontinence) is one of the common complications following ICH, of which impaired gastrointestinal autonomic innervation is a potential mechanism. The incidence of dysphagia after stroke varies depending on screening methods and techniques, with a higher incidence in patients with brainstem (bulbar paralysis) or bilateral hemispheric (pseudobulbar paralysis) lesions.^107^ Dysphagia has been indicated to be an important factor of malnutrition, pneumonia, and death after stroke. The post‐stroke stress ulcer and associated gastrointestinal bleeding often occur in patients with severe neurological deficits, or those with hypothalamus or medulla stroke, which is related to sympathetic hyperactivity and increased local catecholamine, resulting in reduced visceral blood flow and gastrointestinal mucosal ischemia.^108^

### 3.8 Urinary and sexual disturbances

Urinary incontinence and retention are common symptoms after ICH. The physical activity of urination requires the regulation of both autonomic and somatic systems to control the smooth and striated muscle activity of the bladder and urethra. The ICH injury can lead to dysfunction of smooth and striated muscles of the bladder and urethra, leading to urinary incontinence or retention.^9^, ^65^

Sexual dysfunction is another common symptom in ICH patients, and its causes include physiological and psychological factors.^109^ It is indicated that CAN (including the amygdala, paraventricular nucleus, periaqueductal gray, etc.) are involved in maintaining sexual function and penile erection, and brain damage involving these regions is often associated with erectile dysfunction.^9^, ^109^

## 4 ASSESSMENT

### 4.1 HRV

HRV refers to the beat‐to‐beat variation in heartbeat intervals, and the most commonly used HRV analysis is based on the R waves in the continuous electrocardiographic records, calculating the time intervals between adjacent R waves. The HRV can be reflected by time domain analysis, frequency domain analysis, and more sophisticated nonlinear time series analysis.^5^, ^10^, ^52^, ^110^ The time domain analysis of HRV can be represented by different statistical results, as well as visual analysis via the Poincare Plot.^5^, ^52^, ^110^ The frequency domain parameters include high‐frequency (HF; 0.15–0.4 Hz) and low‐frequency (LF; 0.04–0.15 Hz) components. The LF changes are jointly regulated by the sympathetic and parasympathetic nerves, while the HF components are dominated by parasympathetic nerves. The ANS activity can be assessed through the distribution of the LF and HF domain, with LF/HF (the ratio of LF to HF power) reflecting the sympathetic‐parasympathetic balance.^5^, ^52^, ^110^

HRV assessment has attracted increasing interest as a diagnostic tool for detecting autonomic dysfunction and predicting clinical outcome of several cardiac and neurological diseases.^110^ In stroke (including ischemic and hemorrhagic stroke) patients, most observational studies concluded the general decrease of HRV after stroke, with reduced both HF and LF components, which has been demonstrated to be independently associated with adverse outcomes and various complications.^5^, ^7^, ^52^, ^56^, ^95^

### 4.2 BRS

BRS refers to the degree of reflective changes in the cardiac rate caused by changes in arterial blood pressure. BRS is one of the indexes for quantitative analysis of the cardiac autonomic function, reflecting the regulation of the cardiac vagus reflex caused by elevated blood pressure.^111^ BRS can be quantitatively analyzed by the slope of the regression line between the R–R interval (as the abscissa) and systolic blood pressure (as the ordinate), in which a higher slope indicates an enhanced parasympathetic reflex, while a lower slope indicates an enhanced sympathetic reflex.^111^

BRS has been established as a reliable marker of ANS and has been widely used in clinical practice and research.^6^, ^86^ In patients with stroke, the impaired central autonomic centers (especially the insular cortex, hypothalamus, and brainstem) could lead to baroreflex dysfunction. It has been found that BRS was decreased in patients with acute ICH and correlated with increased blood pressure variability, which was demonstrated to independently predict 10‐day short‐term poor outcome.^6^ Also, decreased BRS was indicated to be independently associated with post‐stroke immunosuppression and infections.^86^

### 4.3 Skin sympathetic nerve activity and sympathetic skin response

Skin sympathetic nerve activity (SSNA) is microneurographically recorded from the skin nerve fascicle in the peripheral nerves, which is characterized by burst activity followed by vasoconstriction and/or sweating, and usually elicited by mental stress and arousal stimulation.^112^, ^113^, ^114^ It comprises vasoconstrictor (VC), sudomotor (SM) activity, and vasodilator (VD) activity. The burst amplitude is correlated to both skin blood flow reduction rate and sweat rate change. As a non‐invasive sympathetic activity assessment method, SSNA has been widely used to predict sympathetic tone in cardiac diseases, neurological disorders, sleep apnea, etc.^112^, ^113^, ^114^, ^115^, ^116^ The ICH classification and outcome prediction based on the SSNA signal had been proved to be a feasible method.^114^ Sympathetic skin response (SSR) is also a kind of epidermal potential that is related to sweat gland activity and reflects the function of sympathetic nervous system.^117^, ^118^

### 4.4 Plasma catecholamines

Plasma catecholamine concentration has been widely recognized as an indicator of sympathetic activity in various diseases, including stroke.^9^, ^117^ As a neurotransmitter in the sympathetic postganglionic fibers, the noradrenaline is released into the neuro‐effector junctions (the synaptic gap between the axon terminals and the receptors) when the sympathetic nervous system is activated, and partially penetrates into circulating plasma, increasing the plasma norepinephrine concentration.^9^, ^117^ However, the norepinephrine concentration in circulating plasma may not accurately reflect the real‐time sympathetic activity, because it depends not only on the vesicular release of norepinephrine from sympathetic nerve terminals but also on the capacity of norepinephrine clearance in plasma. In addition, the development of regional norepinephrine spillover rate allows for the evaluation of norepinephrine release from specific organs, which is accepted as the gold standard for quantifying sympathetic activity.^117^, ^119^

## 5 TREATMENT STRATEGIES

### 5.1 Suppression of SNS activity

ICH patients are often complicated with autonomic dysfunction and shift to sympathetic hyperactivity with subsequent increased circulating adrenaline, noradrenaline, and cortisol levels, which is associated with various complications and adverse outcomes mentioned above.^5^, ^6^, ^11^, ^25^, ^29^, ^37^ Accordingly, the application of sympatholytic agents has been proposed as a new therapeutic target in acute stroke to prevent various complications by regulating the sympatho‐parasympathetic balance.^120^, ^121^, ^122^ It has been indicated that BRS, as an indicator of sympathetic overactivity, can be modulated by certain drugs, particularly β‐blockers.^120^, ^121^

As blockers of autonomic neurotransmitter receptors, β‐blockers can also suppress sympathetic hyperactivity after stroke.^14^, ^122^ Laowattana et al. suggested that β‐blockers use was associated with decreased onset severity and better outcomes after acute stroke, which was supposed to be neuroprotective due to a sympatholytic effect associated with decreased thrombin, inflammation, and hemoglobin A1C.^121^ Savitz et al. reported that pre‐treatment with a β‐blocker (carvedilol) before the induction of experimental ischemia resulted in a 40% reduction in infarct volume.^9^ In ICH patients, antihypertensive medications that antagonize the sympathetic nervous system had been indicated to reduce perihematomal edema in a prospective cohort with 303 ICH patients.^123^ In ICH mice, metoprolol treatment had been related to improvement of cardiac and neurological function, through the suppression of sympathetic overactivity.^124^ The β‐blocker use had also been indicated to be associated with a lower risk of intracranial hemorrhage or focal neurological deficit in patients with cerebral cavernous malformation.^14^, ^125^ However, there has been some negative evidence.^126^, ^127^ According to a meta‐analysis comprising 20 studies and more than 100,000 patients with acute stroke (ischemic or hemorrhagic), there was no significant association between β‐blocker therapy and the post‐stroke three main outcomes (mortality, functional outcome, and infections), which did not support the beneficial effects of β‐blocker in the acute phase of stroke.^122^ Therefore, the effect of β‐blocker therapy on ICH outcome needs further investigation. An ongoing multi‐center randomized controlled trial has been investigated to test the safety and efficacy of an β‐blocker (propranolol) in reducing stroke‐associated pneumonia in ICH patients, which may provide a new sight and therapeutic strategy to prevent stroke‐associated pneumonia with early sympatholytic treatment (ClinicalTrials.gov Identifier: NCT05419193). The published and ongoing investigations of ANS modulation with β‐blocker in ICH have been presented in Table 1.

**TABLE 1:** Published and ongoing investigations of ANS modulation in ICH.

Treatment strategies	Authors (year)/Source	Research area	Research design	Objectives (numbers)	Conclusions
β‐blocker	Zhang et al (2021)	Metoprolol therapy and ICH‐induced cardiac damage	Animal experiment	ICH mice model (20–24)	Suppression of sympathetic overactivity by metoprolol attenuates cardiac inflammation, fibrosis, and oxidative stress after ICH
β‐blocker	Sykora et al (2018)	Pre‐admission use of β‐blocker and mortality after ICH	Retrospective analysis	ICH patients (1013)	Pre‐admission use of β‐blocker was not associated with mortality after ICH
β‐blocker	Ongoing (ClinicalTrials.gov Identifier:NCT05419193)	Propranolol therapy and ICH‐associated pneumonia	RCT	ICH patients (100 estimated)	Not yet published
β‐blocker	Terminated (Low enrollement) (ClinicalTrials.gov Identifier:NCT03743103)	Esmolol treatment for hypertension after ICH	RCT	ICH patients (20)	Not yet published
VNS	Hays et al (2014)	VNS and recovery of forelimb motor function after ICH	Animal experiment	ICH rat model (26)	VNS paired with rehabilitative training improves recovery of forelimb function after ICH
VNS	Arsava et al (2022)	Safety and feasibility of nVNS for acute stroke	RCT	Acute stroke patients (61 IS, 8 ICH)	Study supported safety, feasibility, and potential efficacy of nVNS in the setting of acute stroke
VNS	Li et al (2022)	Efficacy and safety of ta‐VNS combined with conventional rehabilitation training in acute stroke	RCT	Acute stroke patients (55 IS, 5 ICH)	The ta‐VNS combined with conventional rehabilitation training for acute stroke is safe and effective
VNS	Ongoing (ClinicalTrials.gov Identifier:NCT04534556)	Safety and feasibility of wireless VNS for chronic stroke	RCT	Chronic (at least 1 year) ischemic or hemorrhagic stroke patients (30 estimated)	Not yet published
VNS	Ongoing (ClinicalTrials.gov Identifier:NCT05771805)	TaVNS and motor functions of upper limb in chronic stroke	RCT	Chronic (3 to 6 month) stroke/TIA patients (50 estimated)	Not yet published
VNS	Ongoing (ClinicalTrials.gov Identifier:NCT02878720)	Combining taVNS and robotic training for recovery of upper limb motor function in chronic stroke	RCT	Chronic (at least 1 year) ischemic or hemorrhagic stroke patients (30 estimated)	Not yet published
VNS	Terminated (funding not continued) (ClinicalTrials.gov Identifier:NCT03292159)	TaVNS for recovery of arm motor function in subacute stroke	RCT	Subacute (4–30 days) ischemic or hemorrhagic stroke patients (6)	Not yet published
VNS	Completed (ClinicalTrials.gov Identifier:NCT04088578)	TaVNS‐supplemented motor retraining in chronic stroke	RCT	Chronic (at least 6 months) ischemic or hemorrhagic stroke patients (26)	Not yet published
VNS	Completed (ClinicalTrials.gov Identifier:NCT04088565)	TaVNS and enhanced motor control in chronic stroke	RCT	Chronic (at least 6 months) ischemic or hemorrhagic stroke patients (26)	Not yet published

Abbreviations: ICH, intracerebral hemorrhage; nVNS, non‐invasive VNS; RCT, randomized controlled trial; taVNS, transcutaneous auricular VNS; TIA, transient ischemic attacks; VNS, vagus nerve stimulation.

### 5.2 Elevation of PNS activity

The vagus nerve is a mixed cranial nerve composed of sensory afferent fibers and motor efferent fibers that transmit sensory and motor information between the central nervous system and autonomic nervous system. Vagus nerve stimulation (VNS) has long been approved for the treatment of refractory epilepsy, primary headache, depression, and obesity.^128^, ^129^ In addition, VNS has been indicated to regulate immune cells and reduce proinflammatory cytokines through cholinergic anti‐inflammatory pathway, thus exerting neuroimmunomodulatory effects in a spectrum of brain disorders.^130^ Recent studies have indicated that VNS paired with rehabilitation is a promising treatment option for people with moderate‐to‐severe neurological impairment after stroke.^131^, ^132^, ^133^, ^134^ The US Food and Drug Administration (FDA) then approved the use of VNS paired with upper limb rehabilitation for the treatment of moderate‐to‐severe upper extremity motor deficits associated with chronic ischemic stroke in 2021.^131^, ^135^ It has also been indicated that VNS during rehabilitative training improved functional recovery after ICH in rats models.^136^ However, due to the invasive nature, side effects, and lack of ready availability, the application of VNS has been limited in clinical trials and practice of ICH.

Consequently, transcutaneous VNS (tVNS) has been developed as a non‐invasive and easily applicable alternative. Transcutaneous cervical VNS (tcVNS) and transcutaneous auricular VNS (taVNS) are currently accepted non‐invasive tVNS modalities in clinical practice and trials.^16^, ^137^, ^138^, ^139^, ^140^, ^141^ The tcVNS is a self‐administrated strategy with a hand‐held stimulation device placed on the neck and was originally developed for the treatment and prevention of episodic cluster headache.^142^ Recent studies have demonstrated the neuroprotective role of tcVNS administration initiated 30 min after transient middle cerebral artery occlusion in rats/mice models, which was supposed to enhance autonomic balance and function, protect the blood–brain barrier integrity, attenuate the infarct volume (approximately 30%), inhibit ischemia‐induced immune activation, and improve the functional outcome without causing significant adverse effects.^15^, ^141^, ^143^ Arsava et al. also demonstrated the safety and feasibility of tcVNS in human patients with ischemic or hemorrhagic stroke within the first 6 h after stroke onset, which suggested that tcVNS may be a promising neuroprotective treatment choice to maintain autonomic balance for acute brain injury, including ICH.^16^, ^137^

Since the vagus nerve distributes to the skin of the external auditory canal and pinna, and vagal reflex responses have been observed upon auricular stimulation, taVNS has also been developed as a non‐invasive tVNS modality for the treatment of various diseases.^138^, ^139^, ^144^, ^145^ Studies had demonstrated the efficacy and safety of taVNS combined with conventional rehabilitation training in acute stroke patients.^145^ In combination with limb rehabilitation training, taVNS may improve coordinated activity of the muscle groups of upper arms by reducing post‐stroke spasticity and increasing motor control.^140^ Studies also indicated that taVNS can improve post‐stroke insomnia and cognitive performance.^146^, ^147^ Animal experiments had demonstrated that taVNS is an effective treatment strategy for patients and model rats with dysphagia after acute stroke.^144^, ^148^ However, to the best of our knowledge, no study has specifically investigated the relationship between taVNS and ICH outcome. The published and ongoing investigations of ANS modulation with VNS in ICH have been presented in Table 1.

## 6 CONCLUSION AND FUTURE PERSPECTIVES

Autonomic dysfunction with CAN damage occurs frequently after ICH and contributes to a series of adverse outcomes. However, the specific mechanism needs to be further clarified, and future structural and functional research on autonomic activities are warranted to better comprehend the underlying mechanisms of autonomic dysfunction after ICH. Additionally, it is imperative to further investigate and demonstrate the relationship between autonomic dysfunction and various types of ICH outcomes in acute and chronic stage. Targeting the autonomic functional activities for risk stratification of patients to establish a more efficient outcome prediction model, thereby achieving more accurate management, is critical to improve the outcomes of ICH patients.

Currently, the management of autonomic dysfunction after ICH lacks specific guidelines or expert consensus, which mainly focuses on symptomatic treatment of various complications. Modulation of the ANS is a promising potential therapeutic strategy for the treatment of post‐ICH autonomic dysfunction. The effects of sympathetic inhibition (especially β‐blockers) and parasympathetic activation (including invasive and non‐invasive VNS) on ICH outcomes need to be further investigated. Also, novel intervention approaches aimed at alleviating sympathetic‐parasympathetic unbalance and randomized controlled trials are necessitated to come out more robust and prospective conclusions.

## FUNDING INFORMATION

This work was supported by the Chinese Academy of Medical Sciences Innovation Fund for Medical Sciences (2019‐I2M‐5‐029) and National Key Research and Development Program of China (2022YFC2504903).

## CONFLICT OF INTEREST STATEMENT

There was no competing interest in the present study.

## Data Availability

Data sharing is not applicable to this article as no new data were created or analyzed in this study.
